# Organoids: physiologically relevant *ex vivo* models for viral disease research

**DOI:** 10.1128/jvi.01132-25

**Published:** 2025-08-29

**Authors:** Yijing Wang, Dingkun Peng, Meilin Li, Meng Yao, Tianlong Li, Su Li, Hua-Ji Qiu, Lian-Feng Li

**Affiliations:** 1 State Key Laboratory for Animal Disease Control and Prevention, Harbin Veterinary Research Institute, Chinese Academy of Agricultural Sciences 687216 , Harbin, Heilongjiang, China; 2 College of Veterinary Medicine, Shanxi Agricultural University 74600, Jinzhong, Shanxi, China; New York University Department of Microbiology, New York, New York, USA

**Keywords:** organoids, viral infections, disease models, stem cells, host-virus interactions

## Abstract

Viral diseases pose serious threats to human health, resulting in substantial economic losses. However, traditional disease models often fail to capture the full complexity of viral pathogenesis. Pluripotent and tissue stem cell-derived organoids help bridge this gap by closely mimicking the structure and function of native organs, thereby enabling new breakthroughs in studying viral pathogenesis. This review discusses the diverse applications of organoid models in virology, including infection modeling, host-virus interaction studies, CRISPR/Cas9-based gene editing, antiviral drug screening, and vaccine development. Here, we focus on human organoid models used to investigate viral infections, covering systemic viral infections (exemplified by viruses such as SARS-CoV-2, Zika virus, influenza virus, and monkeypox virus) as well as localized viral infections (exemplified by viruses including respiratory syncytial virus, herpes simplex virus 1, rotavirus, norovirus, hepatobiliary viruses, and cytomegalovirus). By advancing mechanistic insights and accelerating therapeutic discovery, organoid technology shows significant potential as a complementary tool for combating viral diseases.

## INTRODUCTION

Viral diseases persistently threaten global health systems, causing substantial morbidity, mortality, and socioeconomic burdens. A comprehensive understanding of virus-host interactions is essential for the development of effective antiviral strategies. However, due to the obligate intracellular nature of viruses, traditional research models often fail to accurately replicate the dynamic interplay between viruses and host cells.

Conventional two-dimensional (2D) cell culture, which employs a monolayer growth pattern suitable for studying naturally monolayer-structured tissues, such as intestinal epithelium, fails to recapitulate the physiological microenvironment and functional characteristics of most three-dimensional (3D) tissues. When dissociated from native 3D tissues and subsequently cultured on rigid 2D substrates, cells lose the ability to respond to biochemical gradients that establish cell polarity (1). Since 2D monolayer cultures lack the tissue complexity encountered by pathogens during natural infections, they frequently fail to reliably predict actual infection outcomes (2). Similarly, while animal models are widely employed, their high cost, limited availability, and interspecific differences in viral tropism and immune responses may hinder their ability to fully recapitulate viral pathogenesis (3, 4). These limitations underscore the pressing need for more physiologically relevant models to bridge the gaps between *in vitro* and *in vivo* studies.

Organoids are 3D tissue-like structures derived from stem cells through controlled differentiation, which closely mimic the structural and functional properties of target organs or tissues *in vitro* (5, 6). When embedded in extracellular matrix-mimetic substrates, stem cells self-organize into multicellular spheroids that faithfully recapitulate the structural, transcriptional, and functional features of their native tissue counterparts *in vivo* (7). Unlike conventional models, organoids provide a physiologically relevant system for studying viral infections, enabling long-term virus culture, disease modeling, and detailed analysis of host-virus interactions under controlled conditions (8). This makes them indispensable for investigating viral pathogenesis, screening antiviral drugs, and advancing vaccine development.

This review systematically evaluates the applications of human organoid models in virology, highlighting their roles in both systemic and localized viral infections. Organoid advances are expected to improve these models, offering a powerful tool to study how viruses interact with hosts and aid in the development of new antiviral strategies.

## ORIGINS OF ORGANOID TECHNOLOGY AND KEY MILESTONES IN VIRAL DISEASE RESEARCH

**Fig 1:**
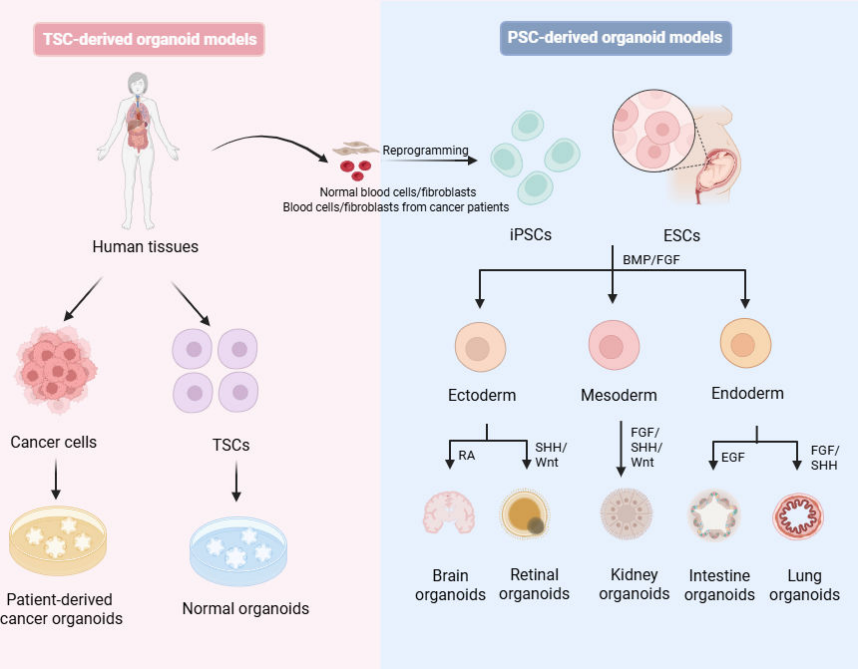
Establishment of pluripotent stem cell (PSC)-derived and tissue stem cell (TSC)-derived organoids. Organoids can be derived from PSCs or TSCs. TSC-derived organoids originate from stem cells in mature organs. When cultured under specific conditions with defined growth factors, these cells undergo self-renewal and differentiate to form organoids that mimic the structure and function of their native tissue. PSC-derived organoids are originate from embryonic stem cells (ESCs) or induced PSCs (iPSCs). iPSCs are generated by reprogramming somatic cells (e.g., blood cells and fibroblasts) using gene editing or transcription factors. These cells possess the capacity to differentiate into cells of the three germ layers (ectoderm, mesoderm, and endoderm), which further differentiate into organ-specific cell types upon appropriate signaling. BMP, bone morphogenetic protein; EGF, epidermal growth factor; FGF, fibroblast growth factor; RA, retinoic acid; SHH, sonic hedgehog; Wnt, wingless-related integration site. Created with https://BioRender.com.

Organoids can be derived from pluripotent stem cells (PSCs), such as embryonic stem cells (ESCs) and induced PSCs (iPSCs), or from tissue stem cells (TSCs) (7, 9). TSC-derived organoids are generated from single TSCs or tissue units like intestinal crypts isolated from postnatal or adult tissues. The culture of these organoids requires specific growth factor cocktails that mimic and sustain the signaling environment necessary for normal tissue homeostasis (10, 11). Patient-derived cancer organoids, a specialized subtype of TSC-derived organoids, are generated directly from cancer stem and progenitor cells isolated from patient tumor tissues, thereby preserving the genomic heterogeneity of the original tumor (12, 13). In contrast, ESC- and iPSC-derived organoids harness stem cell pluripotency through multi-step differentiation protocols. iPSCs are generated by gene editing somatic cells (such as blood cells and fibroblasts) or by reprogramming them using transcription factors (13). These protocols precisely add growth factors or signaling inhibitors to mimic key developmental signals during embryogenesis, such as gastrulation and organogenesis (14). This capacity allows PSCs to generate complex organoids derived from all three germ layers: endoderm, mesoderm, and ectoderm (15) (Fig. 1).

Each source confers distinct characteristics and research value to the resulting organoids. We compare PSC- and TSC-derived organoids, including cellular complexity, maturity, functionality, and tissue architecture (Table 1) (16). PSC-derived organoids model developmental processes, contain multiple cell lineages, and resemble fetal or embryonic stages, but their self-organized structures may differ from those observed *in vivo* (10). In contrast, TSC-derived organoids better reflect the homeostasis, regeneration, or disease state of adult tissues (7). These organoids are usually composed of a single lineage, such as epithelial cells and a few specialized types, showing higher lineage consistency and adult-like functions (17). However, their structures are simpler and they rely on exogenous matrices such as Matrigel to maintain their morphology. These biological, compositional, functional, and structural differences indicate that TSC- and PSC-derived organoids simulate human organs from two dimensions of “adult tissue homeostasis” and “embryonic development,” respectively, and the two are complementary rather than substitutable.

**TABLE 1:** PSC-derived organoids versus TSC-derived organoids

Features	PSC-derived organoids	TSC-derived organoids
Cell source	ESCs or iPSCs	Adult tissue-resident stem/progenitor cells
Modeled process	Development	Adult tissue homeostasis, regeneration, or disease states
Cellular complexity	Multilineage mixtures (endoderm, mesoderm, and ectoderm)	Mainly epithelial cells, supplemented with tissue-specific cells, Single lineage with high lineage uniformity
Functional maturity	Primarily fetal/embryonic functions	Close to adult functions
Organization structure	Self-organized into complex architectures, yet there are differences in spatial arrangement compared with *in vivo*	Simple but uniform structure, relying on exogenous matrices (such as Matrigel) to maintain morphology

**Fig 2:**
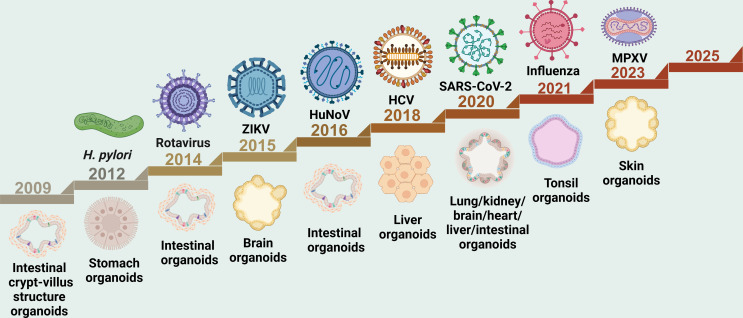
Organoids empowering virology research: timeline of milestone events. Key milestones in the application of organoids to virology research are highlighted. Beginning with the emergence of intestinal organoids featuring crypt-villus architecture in 2009, subsequent developments in stomach organoids, intestinal organoids, and other models established foundational platforms for viral studies. From 2012 to 2023, research on pathogens such as *Helicobacter pylori* (*H. pylori*) (2012), rotavirus (2014), Zika virus (ZIKV) (2015), human norovirus (HuNoV) (2016), hepatitis C virus (HCV) (2018), SARS-CoV-2 (2020), influenza virus (2021), or monkeypox virus (MPXV) (2023) was significantly advanced through diverse organoid models. This progression highlights the evolution of organoid technology from model establishment to broad-spectrum viral pathogen exploration. Created with https://BioRender.com.

Since Clevers’s team first successfully cultured small intestinal organoids from a single *Lgr5*^+^ stem cell in 2009 (18), organoid technology has entered a phase of vigorous development. The establishment of gastric organoids has advanced virus research (19). In 2014, intestinal organoids provided the first direct evidence supporting human enteric virus infection and replication (20). Subsequently, the application of brain organoids in Zika virus (ZIKV) research marked a significant milestone in neurological virus research (21). In 2016, Ettayebi et al. utilized intestinal organoids to achieve *in vitro* infection and transmission of human norovirus (22). As the technology further matured, liver organoids also successfully modeled chronic hepatitis virus infection *in vitro* (23). The outbreak of the coronavirus disease 2019 (COVID-19) pandemic in 2020 greatly accelerated the extensive application of organoid technology, with diverse organoid models—including lung (24), intestine (25), kidney (26), brain (27), and cardiovascular system organoids (28)—rapidly being employed to study viral pathogenic mechanisms. Building upon this progress, tonsil-derived immune organoids were further employed in 2021 to model influenza virus infection, providing novel insights into the evaluation of candidate vaccines (29). More recently, in 2023, researchers successfully established a monkeypox virus (MPXV) infection model using skin organoids, thereby identifying potential therapeutic targets for the virus (30) (Fig. 2). Organoids serve as a key platform for modeling human viral infections, uncovering disease mechanisms, and speeding up the development of new therapies and vaccines.

## APPLICATIONS OF ORGANOIDS IN VIRAL INFECTION RESEARCH

**Fig 3:**
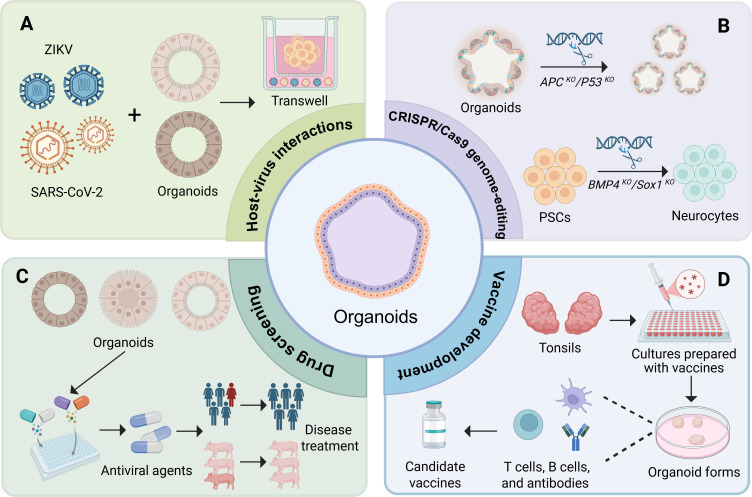
Applications of organoids in viral research. (**A**) Organoids enable the study of infectious diseases through co-culturing with viruses. (**B**) Organoids can be genetically engineered using the CRISPR/Cas9 technology to knockout (KO) cancer-related genes, such as adenomatous polyposis coli (*APC)* and tumor protein p53 (*P53*). Through targeted knockout or activation of specific genes, including transcription factors, CRISPR/Cas9 enables precise regulation of PSC differentiation. For example, disrupting inhibitory genes like bone morphogenetic protein 4 (*BMP4*) or activating neurogenic genes like SRY-box transcription factor 1 (*Sox1*) facilitates neural differentiation. (**C and D**) Organoids further support the development of antivirals and vaccines. Created with https://BioRender.com.

Organoid technology has revolutionized virology research by providing physiologically relevant models that closely mimic the structural and functional characteristics of human organs. These models have been extensively utilized to investigate viral pathogenesis, screen antiviral drugs, and develop vaccines (Fig. 3), demonstrating their potential in understanding viruses and advancing therapies.

### Host-virus interactions

Organoids faithfully recapitulate the cellular diversity and architecture of their originating organs, making them ideal platforms for studying host-pathogen interactions (17). Viral entry typically depends on specific receptors and host cell surface molecules, and organoid models enable the precise investigation of these processes (31) (Fig. 3A). For example, lung organoids can simulate severe acute respiratory syndrome coronavirus 2 (SARS-CoV-2) infection, demonstrating viral replication in airway epithelial and type II alveolar epithelial (AT2) cells while eliciting an immune response (32). Chan et al. utilized nasopharyngeal and bronchial organoids derived from human PSCs to examine host-virus interactions in chronic obstructive pulmonary disease (COPD). Their findings revealed enhanced SARS-CoV-2 replication in COPD-derived organoids, correlating with increased inflammatory responses (33). These studies highlight the value of organoid models in elucidating virus-host interactions at both cellular and molecular levels, providing deeper insights into disease mechanisms (34).

### CRISPR/Cas9 genome-editing in organoids

CRISPR/Cas9, a powerful genome-editing tool derived from bacterial immune systems, enables precise modification of host genes and provides a unique opportunity to study the influence of genetic variations on viral infections. By integrating CRISPR/Cas9 with organoid technology, researchers can systematically assess the roles of specific genes in viral pathogenesis.

Genome-wide CRISPR screens effectively identify host factors for viral infection (Fig. 3B). In coronavirus research, intestinal organoids were employed to generate host gene knockout libraries, confirming angiotensin-converting enzyme 2 (ACE2) and transmembrane protease serine 2 (TMPRSS2) as key host factors in SARS-CoV-2 infection (25, 35). This research also highlighted TMPRSS2 as a potential therapeutic target for broad-spectrum coronavirus treatment (31). In a study focusing on herpes simplex virus 1 (HSV-1), CRISPR/Cas9 targeting the *ICP0* and *ICP27* genes effectively inhibited viral replication and prevented the reactivation of latent infection in brain organoids (36). Collectively, these studies emphasize the physiological relevance of organoid models in recapitulating virus-host interactions and demonstrate the potential of the CRISPR/Cas9 technology for antiviral therapy—whether through targeting host-dependent factors or viral genomes. However, CRISPR/Cas9 applications require careful optimization to minimize off-target effects and cellular stress, and ethical considerations remain crucial, particularly in germline editing.

### Drug screening

Organoids faithfully mimic the complex structure and functions of human organs, providing a powerful platform for antiviral drug screening (Fig. 3C). Organoids not only enable the assessment of drug distribution and delivery efficiency, but also provide more accurate predictions in 3D drug screening than those from traditional 2D cultures (37). Preliminary studies indicate that lung organoid technology holds substantial promise (38). For instance, researchers used human PSC-derived lung organoids infected with SARS-CoV-2 to screen FDA-approved drugs for blocking viral entry (39). Liver organoids recapitulated the complete replication cycle of hepatitis E virus (HEV), enabling high-throughput screening of potent antivirals like brequinar sodium and homoharringtonine sulfate against multiple HEV genotypes, including ribavirin-resistant strains (e.g., G1634R mutant) (40). Organoids accelerate drug discovery by enhancing screening accuracy and efficiency, which is critical for rapidly identifying and validating potential antiviral drugs.

### Vaccine development

Organoid models advance vaccine research by studying viral infections and immune responses. Tonsil organoids assess vaccine immunity, revealing cellular and humoral responses (Fig. 3D). Kastenschmidt et al. compared the immune responses elicited by inactivated influenza vaccine (IIV) and live-attenuated influenza vaccine (LAIV) in human tonsil organoids. Their study revealed that LAIV induced a more robust and diverse immune response compared with IIV (41). Similarly, tonsil organoids have been utilized to assess humoral immune responses following SARS-CoV-2 vaccination (29). These models support antigen-specific somatic hypermutation, affinity maturation, and class switching, which are critical processes for evaluating vaccine efficacy (42). Immune organoids enable personalized vaccine evaluation by modeling human immune responses, reducing animal testing, and accelerating development.

## ORGANOID MODELS FOR VIRAL DISEASE RESEARCH

Viral infections can be classified into systemic and localized types. Systemic infections affect multiple organ systems, causing widespread damage. Examples include SARS-CoV-2 (43), ZIKV (44), influenza virus (45), and MPXV (46). These require multi-organ or organ-on-a-chip models to study dissemination and inter-organ interactions (47). Localized infections target specific tissues, such as respiratory syncytial virus (RSV) in lower respiratory cells (48), HSV-1 in neural tissues (49), rotavirus and norovirus in intestinal cells causing gastroenteritis (50, 51), and hepatitis B (HBV) and C (HCV) viruses in liver (52). Organoid technology enables precise simulation of these viruses' infection, replication, and pathogenic mechanisms.

### Systemic viral infections

#### SARS-CoV-2

SARS-CoV-2 is the causative agent of COVID-19, one of the deadliest pandemics in history. The virus relies on ACE2 as its receptor and requires TMPRSS2 for membrane fusion (53, 54). Initially, the virus targets epithelial cells in the respiratory system, causing hallmark symptoms including cough, mucus production, and dyspnea (42). However, due to widespread ACE2 expression, the infection spreads to the gastrointestinal tract, nervous system, and immune system, thereby triggering hyperinflammation and multi-organ damage (55). Organoids have emerged as valuable tools for investigating SARS-CoV-2 infection, host responses, and drug discovery (Table 2).

**TABLE 2:** Infection characteristics of SARS-CoV-2 in various organoid models^*a*^

Type	Cell source	Cell containing	Tropism	Key factors	Drug screening	Refs.
Nasal organoids	ASCs	Basal cells, ciliated cells, secretory cells, goblet cells, club cells	Ciliated cells	ZO-1, occludin	NA	(56, 57)
Lung organoids	PSCs	AT2 cells	AT2 cells	IL-17, TNF signaling	Imatinib, mycophenolic acid, quinacrine dihydrochloride	(58)
Airway organoids	PSCs, ASCs	AT1 cells, AT2 cells, basal cells, ciliated cells	Ciliated cells, AT2 cells	CIART	Retinoid X Receptor inhibitors HX53, PA452	(59, 60)
Intestinal organoids	PSCs, ASCs	Enterocyte cells, goblet cells, enteroendocrine cells, *LGR5*^+^ stem cells	Enterocytes	IL-1, CXCL8, CXCL11, CTSL	Imatinib, mycophenolic acid, quinacrine dihydrochloride	(58, 61–65)
Cerebral organoids	ESCs, iPSCs	NPCs, neurons, astrocytes, monocytes	Neurons, radial glial cells	Latrophilin-3, bassoon, ISGs	Remdesivir stachel	(66, 67)
Cortical organoids	ESCs	NPCs, neurons, glial cells, choroid plexus cells	Glial cells, choroid plexus cells	Caspase 3	NA	(68)
Islet organoids	ESCs, iPSCs	*β* cells, *α* cells	*β* cells, *α* cells	IFN-*γ*, IL-1*α*	NA	(69)
Liver organoids	iPSCs	Hepatocytes	Hepatocytes	IL-17, TNF, NF-*κ*B	NA	(70)
Trophoblast organoids	PSCs, TSCs	Trophocytes	NA	EGF, TGF-*β*	NA	(71, 72)
Whole-eye organoids	ESCs	Limbal cells, periocular mesenchymal cells, limbal cells, conjunctival cells	Limbal cells	NF-*κ*B, TNF-*α*, types I and III IFNs	NA	(73)
Conjunctiva organoids	ASCs	Keratinocytes, conjunctival tuft cells, goblet cells	Goblet cells	IL-4, IL-13	NA	(74)
Kidney organoids	iPSCs, ESCs	Proximal tubular cells, podocytes, stromal cells, endothelial cells	Proximal tubular cells	ISG15	Remdesivir, LCB1, camostat mesylate, lisinopril, dichloroacetate, human recombinant soluble ACE2	(75–78)
Tonsil organoids	ASCs	Tonsil epithelium cells	Tonsil epithelium cells	ANXA9, ATP6V1C2, LOR, SPTLC3, mRNA	NA	(42, 79)

^*a*^ NA, not applicable.

Nasal organoids have demonstrated that SARS-CoV-2 infects host cells in a two-step process: first, the virus binds to ACE2 receptors on airway ciliated cells; subsequently, ciliary movement transports the virus through the mucus layer (56). Alveolar lung organoids have demonstrated that AT2 cells, which highly express ACE2, are highly susceptible to SARS-CoV-2. Infected cells exhibit characteristics similar to those observed in COVID-19 patients, including the activation of type I/III interferon responses (24, 80–82). To further investigate the heterogeneous responses of lung epithelium, Mulay et al. established a primary human lung epithelial infection model, systematically comparing the initial responses of proximal and distal lung epithelium to SARS-CoV-2 infection (83). Studies using iPSC-derived lung organoids have revealed that SARS-CoV-2 infection alters cellular metabolism by reducing lipid metabolism and increasing glycolysis (9, 84). These models have facilitated therapeutic breakthroughs. For example, Han et al. employed human PSC-derived lung organoids for high-throughput drug screening, identifying imatinib and other effective inhibitors (58), while the viral envelope (E) protein has emerged as a promising therapeutic target (85).

Intestinal organoids closely mimic the cellular composition (e.g., epithelial stem cells and Paneth cells), crypt-villus structure, and functional features of primary intestinal cells (86). Various types have been developed, including small intestinal organoids, colonic organoids (87), and ileum organoids (61). Utilizing these models, research has demonstrated that SARS-CoV-2 not only infects intestinal progenitor cells and epithelial cells (62, 63), but also invades specialized cells, such as enteroendocrine cells and Paneth cells (64). Following infection, viral load increases exponentially and the virus exhibits a dominant apical release pattern (65). Transcriptome analysis reveals high ACE2 expression in mature enterocytes, particularly in duodenal and ileal organoids, while TMPRSS2 and TMPRSS4 synergistically enhance viral infection efficiency (25). Notably, although the proximal and distal intestinal regions exhibit differential responses to SARS-CoV-2 infection, both regions consistently show significant upregulation of interferon-related genes and robust inflammatory responses (62). Importantly, demonstrating the translational utility of this model, studies using iPSC-derived intestinal organoids have shown that the antiviral drug remdesivir effectively inhibits viral replication (64).

Brain organoids research has revealed that SARS-CoV-2 infects choroid plexus cells more efficiently than neurons and astrocytes, a tropism further validated by choroid plexus organoid models (27). Studies demonstrate that in microglia-deficient brain organoids, SARS-CoV-2 infection upregulates synaptic components (66). SARS-CoV-2 infection in brain organoids induces multiple pathological alterations, including microglia-mediated excessive synaptic pruning that disrupts neural circuits (67), astrocyte-facilitated viral propagation (88), and neuronal abnormalities characterized by aberrant tau phosphorylation (89). Blood-brain barrier dysfunction is a key mechanism, as choroid plexus organoid infection impairs the choroid plexus barrier, permitting pathogen and inflammatory factor entry into the brain (90). When integrated into cortical organoids, pericytes form a multicellular complex that enhances viral replication through type I interferon responses (91).

Kidney organoids exhibit approximately twofold higher ACE2 expression in human kidney tubule cells compared with 2D cultures, proving valuable for COVID-19 research (92). SARS-CoV-2 infection of kidney organoids activates pro-fibrotic pathways, potentially leading to fibrosis. Importantly, the diabetic microenvironment (e.g., high glucose fluctuations) increases ACE2 expression in both human kidney organoids and diabetic patient cells (75). This metabolic shift—enhancing glycolysis while suppressing mitochondrial respiration—also heightens susceptibility to SARS-CoV-2 infection (75). Using an optimized proximal tubule organoid model, studies confirmed that kidney cell infection depends on the ACE2 receptor and occurs via two entry pathways: TMPRSS10-mediated surface activation and CTSL/CTSB-dependent endocytosis (76). Critically, these organoids demonstrate that hrsACE2 effectively inhibits SARS-CoV-2 infection by blocking viral entry in kidneys (77).

Studies demonstrate that PSC-derived liver organoids, containing hepatocyte-like and cholangiocyte-like cells, are susceptible targets for SARS-CoV-2 (70, 93). Single-cell analysis provides the first definitive evidence that SARS-CoV-2 directly infects both cell types within PSC-derived liver organoids—a finding corroborated by infection experiments using pseudotyped and live SARS-CoV-2 (70, 94). Infection-induced cell death in cholangiocyte-like cells, compromising biliary barrier function and bile acid transport (95). Meanwhile, hepatocyte-like cells were identified as the primary source of intrahepatic inflammation, activating chemokine and inflammatory pathways that recapitulate the pathological features of hepatic injury observed in clinical COVID-19, with IL-6 signaling identified as the key mechanism for liver-mediated activation of circulating macrophages (70, 94).

Organoid models faithfully recapitulate SARS-CoV-2 infection across the respiratory, digestive, and nervous systems. This high degree of fidelity makes them a crucial platform not only for investigating viral tropism but also for rapidly screening potential therapeutics, such as remdesivir. Furthermore, their capacity to exhibit tissue-specific immune responses and metabolic alterations provides key insights into COVID-19 pathogenesis and supports the development of targeted therapeutic strategies.

#### ZIKV

ZIKV, an enveloped RNA virus belonging to the *Flaviviridae* family, has spread to over 70 countries since emerging in Brazil in 2013 and is linked to neonatal microcephaly (96, 97). Brain organoids effectively model ZIKV tropism, neurodevelopmental impacts, and immune responses (21). Studies demonstrate that ZIKV exhibits strong tropism for neural progenitor cells (NPCs), particularly SOX2^+^ NPCs (21, 98). Infection activates specific signaling pathways and interferon-stimulated genes but induces a weak type I interferon response, potentially impairing cellular function and disrupting brain development (99). While infection of mature cortical organoids may not significantly alter overall size, it increases apoptosis rates and reduces the population of SOX2^+^ NPCs (100). Notably, ZIKV alters DNA methylation patterns of neural genes in brain organoids; these epigenetic changes vary by cell type and are associated with genes involved in neurodevelopment and psychiatric disorders, revealing an additional complex mechanism by which ZIKV disrupts brain development beyond direct infection and immune responses (101). In organoids infected with the attenuated ZIKV-NS2Bᴠ35ᴀ mutant strain, neurotoxicity is significantly reduced, NPC depletion is diminished, and microcephaly-like phenotypes are alleviated (102).

Beyond harming brain development, ZIKV crosses the placenta, where it activates antiviral genes (IFIT/IFITM), disrupts placental models, blocks cell fusion, and impairs development (103). Moreover, the polyphenol desoxyrhapontigenin (DES) potently inhibits ZIKV replication in placental organoids and *in vivo*, significantly reducing vertical transmission and suggesting therapeutic potential against prenatal complications (104).

Brain organoids demonstrate that ZIKV targets NPCs and induces epigenetic dysregulation, while placental organoids clarify its vertical transmission mechanism. These models offer key insights into congenital Zika syndrome and support the evaluation of antiviral drug candidates.

#### Influenza viruses

Influenza viruses cause seasonal epidemics and pandemic threats, with animal strains posing public health risks. It has been shown that the H1N1 subtype infects distal lung organoids through viral receptors (24). Airway organoids, which faithfully recapitulate human airway structures and contain key cell types like goblet, club, and ciliated cells, are susceptible to both human and avian influenza viruses. These models replicate viral tissue tropism and elicit innate immune responses comparable to bronchial explants, validating their utility for respiratory virus research (105). Influenza infection impairs the vital mucociliary clearance defense by reducing ciliary activity and increasing mucus viscosity; mucus flow velocity measurement assesses its impact on airway function (106). Notably, influenza virus strains vary in airway organoid replication efficiency: H1N1pdm and H7N9 replicate well, whereas H5N1 performs poorly, which enables comparison of infectivity potential between strains (107). Researchers have developed large bronchiolar and alveolar organoids expressing the key influenza receptor, sialic acid. Both H5N1 and H7N1 viruses infect these organoids, with H7N1 demonstrating superior replication (108). To enhance physiological relevance, lung endothelial cells were co-cultured, leading to bronchiolar-alveolar lung organoids modeling airway responses. Influenza-infected bronchiolar-alveolar lung organoids show significantly upregulated ICAM-1 expression and robust pro-inflammatory cytokine secretion, closely mirroring viral pneumonia manifestations (109). These advancements provide a robust experimental platform for elucidating the infection mechanisms of influenza viruses and host-virus interactions.

Wagar et al. developed human tonsil organoids using a transwell culture system, successfully generating functional lymphoid structures (29, 110). Crucially, when stimulated with influenza vaccine antigens, this model recapitulates key features of germinal centers *in vitro*, including hemagglutinin-specific antibody production, somatic hypermutation, affinity maturation, plasma cell differentiation, and class-switch recombination, thereby providing a platform to dissect the cellular mechanisms underlying influenza vaccine responses (41). Subsequently, Kastenschmidt et al. compared the adaptive immune responses elicited by IIV, LAIV, and H1N1 influenza virus in this organoid model (41). Comprehensive immunologic analysis further identified the frequency of Th1 cells in host lymphoid tissues as a key predictor of IIV-induced neutralizing antibody responses (111).

Respiratory organoids recapitulate the tissue tropism of influenza viruses and impaired mucociliary clearance, while tonsil organoids reveal differences in vaccine-induced immune responses. Taken together, these models enhance the understanding of viral pathogenesis and accelerate the evaluation of influenza vaccines.

#### MPXV

MPXV, belonging to the *Orthopoxvirus* genus of the *Poxviridae* family, primarily causes skin lesions but can also lead to systemic manifestations such as diarrhea, neurological dysfunction, and respiratory complications (112). To investigate MPXV susceptibility, studies show that the virus efficiently infects kidney organoids, causing structural damage, with electron microscopy capturing the complete viral assembly from crescents to mature virions (113). In colon organoids, Clade I MPXV infection downregulates zinc homeostasis-related genes (e.g.*,* SLC30A2 and MT1G). Notably, a machine learning model based on key genes (e.g.*,* TFF1 and HSPA6) identified through Cross-RMAS analysis of RNA-seq data achieved 100% accuracy in distinguishing between different MPXV clades and mock-infected controls (114, 115). Furthermore, iPSC-derived multilayered skin organoids, which incorporate structures like hair follicles, support the complete MPXV life cycle. Electron microscopy reveals an assembly process similar to that of other poxviruses, uncovering the molecular basis for MPXV-induced skin damage: the virus downregulates genes involved in skin development and keratinization, compromising the epidermal barrier, activating inflammatory responses, and inducing cytoskeletal remodeling (30). The FDA-approved antiviral drug tecovirimat, targeting the viral envelope protein VP37, inhibits the wrapping process required for infectious virion release from host cells, thereby suppressing productive infection (30).

These studies reveal MPXV’s replication mechanisms, pathogenic features, and host responses in key organs, confirm the efficacy of antiviral drugs, and emphasize the critical role of organoid models integrated with multi-omics and AI in elucidating viral pathogenesis and accelerating the development of prevention and treatment strategies.

### Localized viral infections

#### RSV

RSV infects individuals of all ages but is particularly severe in infants under six months, causing tens of thousands of deaths annually, especially in resource-limited regions (87, 116). Nearly all children are infected by age of 2, with lifelong reinfections possible (117). Infants are more vulnerable due to their narrower airways, which are easily obstructed by mucus secretions from the pseudostratified epithelium (118). The lack of clinically relevant models has hindered the understanding of RSV pathogenesis and the development of therapies.

Rajan et al. developed a human nasal organoid-based model using non-invasive techniques, preserving the structure of the respiratory epithelium (119). When differentiated at the air-liquid interface, human nasal organoids form a pseudostratified epithelium containing basal, goblet, club, and ciliated cells (119). Infection of this model with RSV (including contemporary strains, RSV/A/ON and RSV/B/BA) recapitulates key features of infection, including viral shedding, ciliary damage, innate immune responses, and mucus hypersecretion. Infection occurs primarily at the apical surface, but stronger basolateral cytokine responses suggest signal translocation (120). The neutralizing monoclonal antibody palivizumab, targeting the RSV F protein (106), suppresses infection in a dose-dependent manner when administered basolaterally (119). Airway organoids cultured at the air-liquid interface further model the human airway environment and remain susceptible to the co-circulating RSV subgroups A and B (121). Despite predominant type III interferon responses, RSV replicates efficiently in both upper and lower airway cells (subgroup A showing a slight advantage), and this replication leads to cytopathic effects such as rounding of ciliated cells, cilia disruption, and syncytia formation (122). Significantly, within this airway organoids model, inhibition of protein kinase C zeta (PKC*ζ*) activation prevented nucleolin trafficking to RSV particles and reduced viral replication and pathology, findings corroborated in the RSV-infected mice (123). This model supports the study of infant susceptibility and the development of novel antiviral strategies.

#### HSV-1

HSV-1 is a human-specific virus that establishes lifelong latency primarily in the sensory neurons of the trigeminal ganglia (124). Krenn et al. showed HSV-1 induces microcephaly in brain organoids by suppressing cell proliferation/differentiation, leading to structural damage (99). D'Aiuto et al. successfully modeled the HSV-1 infection process, including acute infection, latency, and reactivation, using 2D and 3D culture models derived from human iPSCs (125). This infection model replicates neurodegenerative pathologies (e.g., cell death and gliosis) and notably triggers amyloid beta (A*β*) deposition, massive neuronal loss, reactive gliosis, and neuroinflammation—changes closely resembling hallmarks of Alzheimer’s disease (AD), suggesting HSV-1 may contribute to AD pathogenesis (126). Organoid infection models also reveal enhanced inflammatory responses (elevated pro-inflammatory cytokines TNF-*α*/IL-6/IL-10/IL-4 and immune cell infiltration). The drugs ribavirin and valaciclovir effectively inhibit HSV-1 replication and alleviate neuropathological symptoms (126). These findings not only deepen the understanding of the neuropathogenic mechanism of HSV-1 but also successfully establish organoid models that can simulate the complete infection cycle, thereby laying a solid experimental foundation for subsequent mechanism research and drug screening.

#### Rotaviruses

Rotaviral infection remains the leading cause of severe dehydrating gastroenteritis in children. Although rotavirus vaccination was introduced globally more than a decade ago, approximately 200,000 deaths still occur each year (50). Since their establishment in 2012, intestinal organoids have proven superior to traditional monkey kidney epithelial cell lines (e.g., MA104) in rotavirus studies. Notably, intestinal organoids support efficient replication of clinical rotavirus isolates directly obtained from fecal samples, with most strains achieving viral titers up to 10-fold higher than those in conventional cell cultures. Viral replication was unexpectedly detected in both epithelial and mesenchymal compartments of intestinal organoids, suggesting a broader tropism of rotavirus than ever recognized (127). Subsequent studies have shown that intestinal organoids are an effective model for studying the biology of human rotavirus (HRV) and can simulate multiple characteristics of HRV infection, including host range limitation, cell type preference, virus-induced fluid secretion, and vaccine strain replication differences (128). Chang-Graham et al. further elucidated that rotavirus-infected cells release ADP to activate P2Y1 receptors on neighboring cells, inducing intercellular calcium waves that amplify viral replication, inflammatory cytokine release, and intestinal fluid secretion, ultimately driving diarrhea pathogenesis (129). In 2023, Zhang et al. verified that metformin hydrochloride significantly inhibited rotavirus replication in intestinal organoids and confirmed the feasibility of organoids as a model for *in vitro* viral infection (130). These advancements underscore the critical role of intestinal organoids in elucidating novel pathogenic mechanisms of rotavirus and expediting antiviral drug development, thereby offering a vital research platform for reducing mortality associated with rotavirus infection in children.

#### Norovirus

Human norovirus (HuNoV) is the most common pathogen responsible for epidemic sporadic acute gastroenteritis worldwide and a major cause of foodborne outbreaks (51, 131). Previous efforts to culture HuNoV in intestinal cell lines and primary immune cells failed, limiting research on virus-host interactions due to the absence of reliable *in vitro* systems (132). A breakthrough came when Ettayebi et al. successfully cultured multiple HuNoV strains, such as GII.4, in stem cell-derived intestinal organoids monolayer cultures. Both GII.4 and GII.3 HuNoV replicate in intestinal organoids of duodenal, jejunal, and ileal origins. However, replication of some strains depends on bile, which acts on host cells rather than the virus itself. For these strains, bile must be added during or after viral adsorption to enable replication (22). Subsequent studies revealed that interferon responses inhibit HuNoV replication in intestinal epithelial cells, and suppressing this response via gene editing or drugs promotes viral replication (133). The efficiency of HuNoV infection varies depending on culture media and the sources of intestinal organoids. Ettayebi et al. found that the intestinal organoids growth medium significantly enhances viral replication. Additionally, the susceptibility of different intestinal organoids to infection depends on multiple factors. Through systematic optimization, the team expanded the range of cultivable HuNoV strains and refined the culture system (134). Intestinal organoids have overcome the challenge of norovirus *in vitro* culture, revealing the replication features of bile-dependent strains and the antiviral effects of interferon. This advancement establishes a foundation for investigating norovirus-host interactions and optimizing culture systems.

#### Hepatitis viruses

The liver is a highly vascularized organ constantly exposed to blood-borne pathogens (135). HBV and HCV, the common pathogens of viral hepatitis, disrupt hepatic cellular homeostasis, cause liver dysfunction, and may progress to liver fibrosis or cirrhosis (136, 137). The 3D liver organoids expressing viral-localizing molecules developed by Baktash et al. uncovered HCV invasion *via* single-molecule imaging: an actin-dependent process initially engaging basolateral receptors (SR-B1/CD81/EGFR), then migrating to tight junctions to bind CLDN1/OCLN, and ultimately undergoing internalization via EGFR-dependent clathrin-mediated endocytosis (138). Furthermore, Lee et al.’s co-culture model of macrophages and liver organoids generated liver-resident Kupffer-like cells, demonstrating that HCV triggers lipogenesis and lipid accumulation—enhanced by macrophages. In contrast, prolonged fatty acid exposure increased HCV replication, inflammation, and fibrosis, highlighting a harmful viral-metabolic cycle (139).

Recent progress in organoid technology has greatly advanced HBV research. IPSC-derived liver organoids outperform hepatocyte-like cells in liver function and express elevated HBV receptor sodium taurocholate cotransporting polypeptide (NTCP). Following HBV infection, liver organoids exhibit significantly higher levels of pregenomic RNA, intracellular viral DNA (vDNA), covalently closed circular DNA (cccDNA), and supernatant vDNA compared to iPSC-derived hepatocyte-like cells. Concurrently, HBV-infected organoids demonstrate reduced expression of liver-specific genes, elevated hepatic failure markers, and structural damage (23). These reliably model HBV infection and injury, enabling therapeutic development. De Crignis et al. further demonstrated that both healthy donor- and patient-derived organoids support *in vivo*-like HBV replication, with differentiated organoids efficiently producing cccDNA, HBeAg, viral RNA and proteins, and infectious particles (140). This platform facilitates antiviral screening, for example, tenofovir and fialuridine inhibiting HBV DNA, while organoids accurately model fialuridine hepatotoxicity (140). These achievements highlight the core value of organoids in elucidating the pathogenic mechanisms of liver viruses and advancing the development of targeted therapies.

#### Cytomegalovirus

Human cytomegalovirus (HCMV) causes congenital central nervous system disorders including hearing loss, cognitive impairment, and microcephaly (141). To investigate its impact on brain development, Sun et al. employed iPSC-derived brain organoids, demonstrating that clinical HCMV strains disrupt organoid structure and neural network activity (142). Specifically, infection induces necrosis, vacuolation, and cyst formation, impairing neurodevelopment via reduced cortical sites, disrupted tissue lamination, and abnormal neural marker *β*-tubulin III expression—effects preventable by neutralizing antibodies (143). NPCs, pivotal for cortical expansion and synaptogenesis, rely on Ca²^+^ signaling to regulate differentiation and intercellular communication (144). HCMV infection dysregulates Ca²^+^ dynamics in NPCs and organoids, promoting viral spread and neural impairment (145). The mechanism of HCMV-induced hearing loss remains unclear. Studying human inner ear cells is challenging due to bony encapsulation. Harding et al. demonstrated that HCMV-infected, stem cell-derived ear progenitors show no interferon/inflammatory response, suggesting viral disruption of developmental pathways (143). Brain and inner ear organoid studies have uncovered how HCMV disrupts neural development and demonstrated the protective efficacy of neutralizing antibodies, offering a crucial model for studying congenital neural damage.

## CONCLUDING REMARKS

Organoids have transformed viral research, providing new insights into infections and therapies. Here, we systematically summarize the applications of organoid technology in viral research, highlighting its advantages and limitations in mimicking organ structures and functions, studying host-virus interactions, drug screening, vaccine development, and disease modeling. These models have provided unprecedented opportunities to unravel viral infection mechanisms, host immune responses, and antiviral drug development.

However, the translational value of organoid technology in clinical predictive applications is limited by its inability to precisely simulate the *in vivo* physiological environment, and the resulting discrepancy between experimental data and actual clinical outcomes constitutes a key translational bottleneck. Current organoid systems face multiple challenges: in terms of cell composition, TSC-derived organoids lack stromal cells and complete functional networks, leading to abnormal microenvironments and signaling pathways; iPSCs encounter technical obstacles in differentiation and, due to short culture periods, are unable to fully mature. Additionally, traditional models generally lack immune cells and functional vascular systems. The absence of immune components affects viral clearance and immune pathology modeling (146, 147), while the absence of vascular systems not only causes tissue necrosis (57) and hinders immune and viral spread, but its integration remains a persistent challenge. To address the immune system deficiency, strategies such as integrating autologous tissue-resident memory T cells into epithelial organoids are being developed (146). This approach can form *in vivo*-like mucosal immune structures, providing a new platform for intestinal immune modeling.

In terms of multi-organoid function simulation, single-organ systems cannot replicate the drug metabolism or virus transmission processes within the body. Drug metabolism involves multiple organs (such as the liver and kidneys) and multiple stages (absorption, transformation, and excretion). Single-organ systems only assess the impact of drugs on one organ in isolation and cannot simulate the overall drug metabolism. This leads to inaccurate pharmacodynamic evaluations and limits the prediction of drug efficacy and safety. Additionally, these systems cannot replicate the transmission of viruses via the bloodstream, lymphatic system, or other dissemination routes.

During the process of industrial application, organoid technology faces the severe challenge of high-throughput screening. Due to their reliance on specialized matrices and long-term culture, organoids are difficult to adapt to the 96-/384-well format high-throughput screening system. Moreover, there are problems of poor reproducibility and low standardization during the culture process, which limit their application in large-scale drug screening.

To break through these predicaments, cutting-edge research is focusing on the development of multi-organ-on-a-chip systems, which connect lung, liver, lymphoid, and other organoid modules through microfluidic channels and combine with physiology-based pharmacokinetic modeling to dynamically track viral transmission and drug metabolism, initially demonstrating the potential to simulate complex *in vivo* physiological processes. In the future, cross-platform data integration, multi-dimensional functional validation, and iterative dynamic model optimization will further enhance the predictive value of organoids for clinical outcomes. Meanwhile, the deep integration of automated imaging and artificial intelligence-driven analysis is expected to build a next-generation *in vitro* antiviral drug screening platform with stronger predictive capabilities, providing more reliable *in vitro* model support for the study of viral pathogenic mechanisms.

Despite limitations, organoid technology remains critical to viral research, expanding across disciplines to bridge basic science and clinical translation. Integration with emerging technologies like AI, multi-omics, and single-cell sequencing will enhance its impact. Through interdisciplinary collaboration and innovation, organoid systems can advance viral research and support precision medicine and infectious disease management.
